# WSES Jerusalem guidelines for diagnosis and treatment of acute appendicitis

**DOI:** 10.1186/s13017-016-0090-5

**Published:** 2016-07-18

**Authors:** Salomone Di Saverio, Arianna Birindelli, Micheal D. Kelly, Fausto Catena, Dieter G. Weber, Massimo Sartelli, Michael Sugrue, Mark De Moya, Carlos Augusto Gomes, Aneel Bhangu, Ferdinando Agresta, Ernest E. Moore, Kjetil Soreide, Ewen Griffiths, Steve De Castro, Jeffry Kashuk, Yoram Kluger, Ari Leppaniemi, Luca Ansaloni, Manne Andersson, Federico Coccolini, Raul Coimbra, Kurinchi S. Gurusamy, Fabio Cesare Campanile, Walter Biffl, Osvaldo Chiara, Fred Moore, Andrew B. Peitzman, Gustavo P. Fraga, David Costa, Ronald V. Maier, Sandro Rizoli, Zsolt J Balogh, Cino Bendinelli, Roberto Cirocchi, Valeria Tonini, Alice Piccinini, Gregorio Tugnoli, Elio Jovine, Roberto Persiani, Antonio Biondi, Thomas Scalea, Philip Stahel, Rao Ivatury, George Velmahos, Roland Andersson

**Affiliations:** Emergency and Trauma Surgery – Maggiore Hospital, AUSL, Bologna, Italy; S. Orsola Malpighi University Hospital – University of Bologna, Bologna, Italy; Locum Surgeon, Acute Surgical Unit, Canberra Hospital, Canberra, ACT Australia; Emergency and Trauma Surgery Department, Maggiore Hospital of Parma, Parma, Italy; Trauma and General Surgeon Royal Perth Hospital & The University of Western Australia, Perth, Australia; Macerata Hospital, Macerata, Italy; Letterkenny Hospital, Donegal, Ireland; Harvard Medical School - Massachusetts General Hospital, Boston, USA; Department of Surgery Hospital Universitario, Universidade General de Juiz de Fora, Juiz de Fora, Brazil; Academic Department of Surgery, University Hospitals Birmingham NHS Foundation Trust, Edgabaston, Birmingham, UK; General Surgery, Civil Hospital - ULSS19, Veneto, Adria, RO Italy; Denver Health System – Denver Health Medical Center, Denver, USA; Department of Gastrointestinal Surgery, Stavanger University Hospital, Stavanger, Norway; University Hospitals Birmingham NHS Foundation Trust Queen Elizabeth Hospital, Birmingham, UK; Department of Surgery, OLVG, Amsterdam, The Netherlands; Department of Surgery, University of Jerusalem, Jerusalem, Israel; Division of General Surgery, Rambam Health Care Campus, Haifa, Israel; Abdominal Center, University of Helsinki, Helsinki, Finland; General Surgery I, Papa Giovanni XXIII Hospital, Bergamo, Italy; Department of Surgery, Linkoping University, Linkoping, Sweden; UCSD Health System - Hillcrest Campus Department of Surgery Chief Division of Trauma, Surgical Critical Care, Burns, and Acute Care Surgery, San Diego, CA USA; Royal Free Campus, University College London, London, UK; Department of Surgery, San Giovanni Decollato Andosilla Hospital, Viterbo, Italy; Queen’s Medical Center, University of Hawaii, Honolulu, HI USA; Niguarda Hospital, Milan, Italy; University of Florida, Gainesville, USA; Department of Surgery, University of Pittsburgh School of Medicine, UPMC-Presbyterian, Pittsburgh, USA; Faculdade de Ciências Médicas (FCM) - Unicamp, Campinas, SP Brazil; Alicante, Spain; Department of Surgery, University of Washington, Harborview Medical Center, Seattle, WA USA; St. Michael Hospital, Toronto, Canada; Department of Traumatology, John Hunter Hospital and University of Newcastle, Newcastle, NSW Australia; Department of Surgery, Terni Hospital, University of Perugia, Terni, Italy; Trauma Surgery Unit - Maggiore Hospital AUSL, Bologna, Italy; Department of Surgery, Maggiore Hospital AUSL, Bologna, Italy; Catholic University, A. Gemelli University Hospital, Rome, Italy; Department of Surgery, University of Catania, Catania, Italy; R. Adams Cowley Trauma Center, Baltimore, MD USA; Professor Emeritus Virginia Commonwealth University, Richmond, VA USA; Harvard Medical School - Chief of Trauma, Emergency Surgery, and Surgical Critical Care, Massachusetts General Hospital, Boston, USA

**Keywords:** Acute Appendicitis, Guidelines, Consensus Conference, Alvarado Score, Appendicitis diagnosis score, Non-operative management, Antibiotics, Complicated appendicitis, Appendectomy, Laparoscopic appendectomy, Phlegmon, Appendiceal abscess

## Abstract

Acute appendicitis (AA) is among the most common cause of acute abdominal pain. Diagnosis of AA is challenging; a variable combination of clinical signs and symptoms has been used together with laboratory findings in several scoring systems proposed for suggesting the probability of AA and the possible subsequent management pathway. The role of imaging in the diagnosis of AA is still debated, with variable use of US, CT and MRI in different settings worldwide. Up to date, comprehensive clinical guidelines for diagnosis and management of AA have never been issued. In July 2015, during the 3rd World Congress of the WSES, held in Jerusalem (Israel), a panel of experts including an Organizational Committee and Scientific Committee and Scientific Secretariat, participated to a Consensus Conference where eight panelists presented a number of statements developed for each of the eight main questions about diagnosis and management of AA. The statements were then voted, eventually modified and finally approved by the participants to The Consensus Conference and lately by the board of co-authors. The current paper is reporting the definitive Guidelines Statements on each of the following topics: 1) Diagnostic efficiency of clinical scoring systems, 2) Role of Imaging, 3) Non-operative treatment for uncomplicated appendicitis, 4) Timing of appendectomy and in-hospital delay, 5) Surgical treatment 6) Scoring systems for intra-operative grading of appendicitis and their clinical usefulness 7) Non-surgical treatment for complicated appendicitis: abscess or phlegmon 8) Pre-operative and post-operative antibiotics.

## Background

Acute appendicitis (AA) is a common cause of acute abdominal pain, which can progress to perforation and peritonitis, associated with morbidity and mortality. The lifetime risk of appendicitis is 8.6 % for males and 6.7 % for females; however, the risk of undergoing appendectomy is much lower for males than for females (12 vs. 23 %) and it occurs most often between the ages of 10 and 30, with a male:female ratio of approximately 1.4:1 [1]. Despite numerous studies on AA, many unresolved issues remain, including aetiology and treatment. The diagnosis of AA is a constellation of history, physical examination coupled with laboratory investigations, supplemented by selective focused imaging. These can be used in combination in scoring systems. Various clinical scoring systems have been proposed in order to predict AA with certainty, but none has been widely accepted. The role of diagnostic imaging (ultrasound (US), computed tomography (CT) or magnetic resonance imaging (MRI)) is another major controversy.

The surgical treatment of AA has undergone a paradigm shift from open appendectomy to laparoscopic appendectomy, both in adults and now also in paediatric cases. Over the last decade non-operative treatment with antibiotics has been proposed as an alternative to surgery in uncomplicated cases [2], while the non-surgical treatment played an important role in the management of complicated appendicitis with phlegmon or abscess [3]. Another major issue in management still open to debate is the timing of appendectomy and the safety of in-hospital delay. Moreover, there are debated recommendations on the type of surgical treatment and the post-operative management including antibiotic therapy.

For these reasons the World Society of Emergency Surgery (WSES) decided to convene a Consensus Conference (CC) to study the topic and define its guidelines regarding diagnosis and treatment of AA.

## Material and methods: organizational model

On August 2013 the Organizational Board of the 2^nd^ World Congress of the World Society of Emergency Surgery (WSES) endorsed its president to organize the Consensus Conference (CC) on AA in order to develop WSES Guidelines on this topic. The WSES President appointed four members to a Scientific Secretariat, eight members to an Organizational Committee and eight members to a Scientific Committee, choosing them from the expert affiliates of the Society. Eight key questions on the diagnosis and treatment of AA were developed in order to guide analysis of the literature and subsequent discussion of the topic (Table 1). Under the supervision of the Scientific Secretariat, a bibliographic search related to these questions was performed through April 2015 without time or language restriction. The key words used for the electronic search are listed in Table 1. Additionally a manual literature search was performed by each of the members of the working groups involved in the analysis of the above-mentioned eight questions. Prior to the Consensus Conference, a number of statements were developed for each of the main questions, along with the Level of Evidence (LoE) and the Grade of Recommendation (GoR) for each statement. The 2011 Oxford Classification was used to grade the LoE and GoR. Provisional statements and their supporting evidence were then submitted for review to all the participating members of the Consensus Conference and to the WSES board members by email before the Conference. Modifications were performed when necessary based on feedback.

**Table 1:** Key questions and key words used to develop the Consensus Conference on Acute Appendicitis (AA)

Key questions	Key words
1. Diagnostic efficiency of clinical scoring systems *Diagnostic efficiency of clinical scoring systems and their role in the management of patients with suspected appendicitis - can they be used as basis for a structured management?*	Derivation OR clinical OR predict OR decision AND rule OR algorithm OR tool OR model OR score OR indicator OR validation OR criteria AND appendicitis
2. Role of imaging *Routine vs selective imaging? CT or US or both? In what order?*	Diagnosis OR imaging AND selective OR routine AND ultrasound OR computed AND tomography OR US OR CT OR MRI AND adult OR child OR pregnant AND appendicitis
3. Non-operative treatment for uncomplicated appendicitis. *What is the natural history of appendicitis? Can appendicitis resolve without treatment? How common is it?*	Uncomplicated AND appendicitis AND pathogenesis OR antibiotics OR nonoperative OR conservative OR spontaneous AND resolution or self-limiting AND treatment OR management
4. Timing of appendectomy and in-hospital delay *Does in-hospital delay increasethe rate of complication or perforation? Is it safe to delay appendectomy? Timing of appendectomy*	Appendectomy AND delay OR perforation OR complication OR indicator OR criteria AND appendicitis
5. Surgical treatment *-open or laparoscopic?* *-lavage or aspiration of pus?* *-drains?* *-ligation or invagination of the stump?* *-primary or secondary closure of the wound?*	Surgery OR operative OR laparoscopy OR open OR treatment OR management AND elder OR comorbidities OR obese OR child OR pregnant AND complicated OR perforated OR abscess AND lavage OR aspiration OR suction OR drain OR mesoappendix OR sealing OR monopolar OR bipolar OR staple OR endoloop OR stump OR invagination OR ligation AND appendicitis
6. Scoring systems for intra-operative grading of appendicitis and their clinical usefulness *What are the histopathological criteria for appendicitis of clinical importance? Minor inflammatory changes, early appendicitis, catarrhal appendicitis. The criteria used will have an influence on the proportion of negative appendectomy, and also on evaluation of diagnostic performance.*	intra-operative AND grade OR score OR indicator OR criteria AND histopathology OR macroscopic AND diagnosis OR surgeon AND experience AND appendicitis
7. Non-surgical treatment of complicated appendicitis: abscess or phlegmone *Role of percutaneous drainage and Interval Appendectomy or immediate surgery.*	Abscess OR phlegmon AND drain OR percutaneous OR interval AND appendectomy AND conservative OR nonsurgical AND treatment OR management AND complicated AND appendicitis
8. Preoperative and Postoperative Antibiotics *Should Preoperative antibiotics prophylaxis be given? What antibiotics? When should postoperative antibiotics be given? What antibiotics? Duration?*	Antibiotic OR antimicrobial OR infection OR prophylaxis OR therapy OR treatment AND appendectomy OR surgery AND time OR day OR range OR duration AND complicated OR uncomplicated AND intravenous OR oral AND appendicitis

**Fig. 1:**
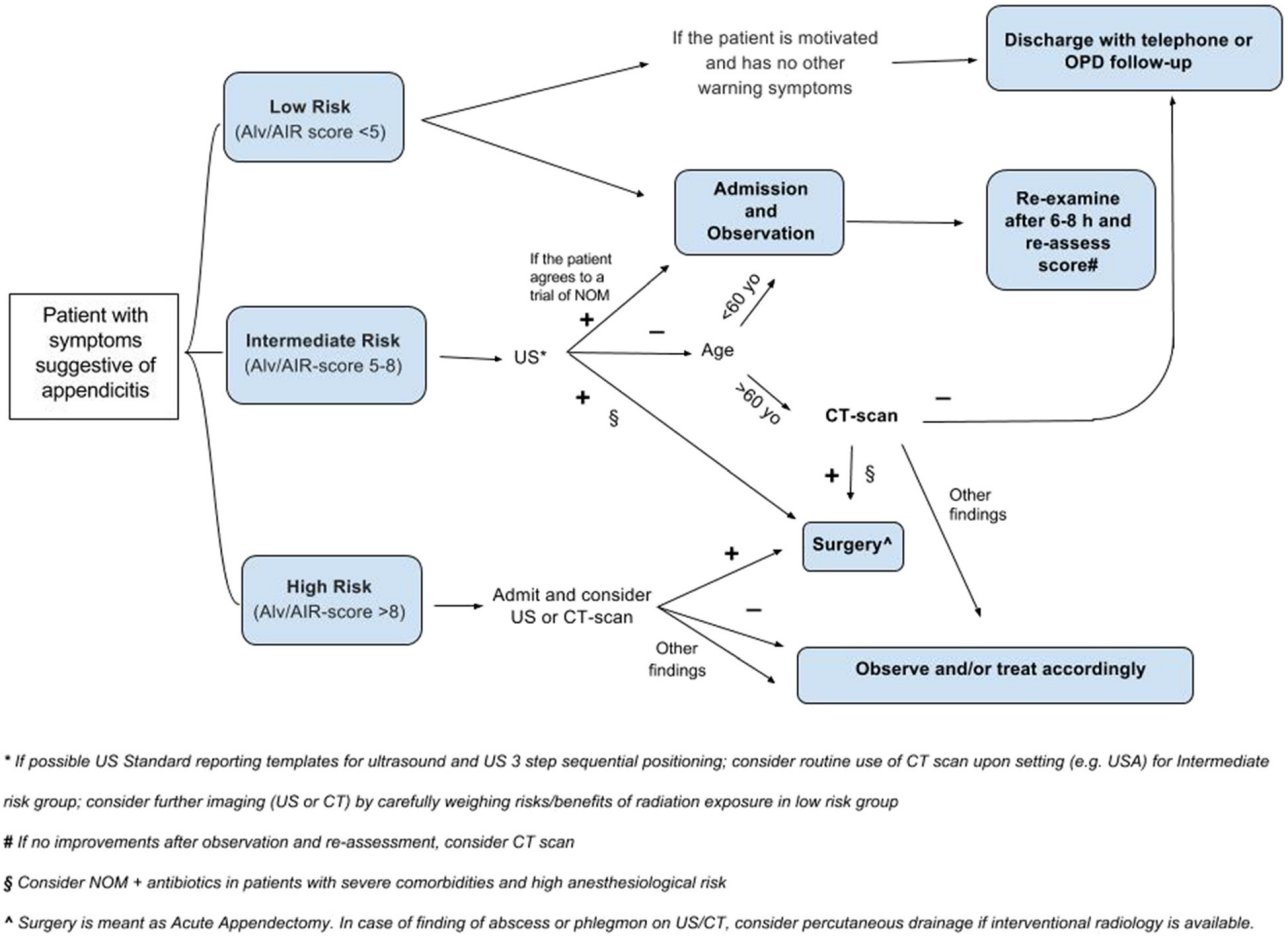
Practical WSES algorithm for diagnosis and treatment of patients with suspected acute appendicitis

The Consensus Conference on AA was held in Jerusalem, Israel, on July 6th, 2015 during the 3rd World Congress of the WSES. During the first part of this CC, a member of each group (S. Di Saverio, M.D. Kelly, D. Weber, F. Catena, M. Sugrue, M. Sartelli, M. De Moya, C.A. Gomes) presented each of the statements along with LoE, GoR, and the literature supporting each statement. Each statement was then voted upon by the audience in terms of “agree” or “disagree” using an electronic voting system. The percentage of agreement was recorded immediately; in case of greater than 30 % disagreement, the statement was modified after discussion. Furthermore, comments for each statement were collected in all cases. Before the second part of the Consensus Conference, the president and representatives from the Organizational Committee, Scientific Committee and Scientific Secretariat modified the statements according to the findings of the first session of the CC. The revised statements were then presented again to the audience. During the Consensus Conference, a comprehensive algorithm for the treatment of AA was developed based on the results of the first session of the CC and voted upon for definitive approval (Fig. 1). The final statements, along with their LoE and GoR, are available in Appendix. All statements are reported in the following Results section, subdivided by each of the eight questions, with the relative discussion and supportive evidence.

## Results

### Diagnostic efficiency of clinical scoring systems

*Diagnostic efficiency of clinical scoring systems and their role in the management of patients with suspected appendicitis - can they be used as basis for a structured management?(Speaker in Jerusalem CC Dr. D. G. Weber)*

Multiple diagnostic scoring systems have been developed with the aim to provide clinical probabilities that a patient has acute appendicitis. These scores typically incorporate clinical features of the history and physical examination, and laboratory parameters. Most popular and validated examples include the Alvarado score (also known as the MANTRELS score) [4], the Paediatric Appendicitis Score (PAS) [5], the Appendicitis Inflammatory Response (AIR) Score [6], the Raja Isteri Pengiran Anak Saleha Appendicitis (RIPASA) score [7] and, most recently, the Adult Appendicitis Score (AAS) [8]. A comparison among these clinical scores is reported in Table 2. Alvarado and AIR scores are currently the most often used scores in the clinical settings. The primary data from which these scores have been derived are largely from retrospective and prospective cross-sectional studies, and represent either level 2 or 3 evidence.

**Table 2:** Comparison of the most popular and validated clinical scores for the diagnosis of AA

	Alvarado score^a^	AIR score^b^	PAS score^c^	RIPASA score^d^	AAS score^e^
Vomiting		1			
Nausea or vomiting	1		1	1	
Anorexia	1		1	1	
Pain in RIF^f^	2	1		0.5	2
Migration of pain to the RIF^f^	1		1	0.5	2
Rovsing’s sign				2	
RIF^g^ tenderness			2	1	
Women >50 years or men (any age)					3
Women <50 years					1
Rebound tenderness or muscular defense/guarding	1			1 + 2	
Light		1			2
Medium		2			4
Strong		3			4
Body temperature					
> 37.5 °C	1		1		
> 38.5 °C		1			
> 37– <39 °C				1	
WBC (white blood cell) count					
> 10.0 × 10^9^/l	2		1	1	
10.0–14.9 × 10^9^/l		1			
≥ 15.0 × 10^9^/l		2			
≥ .2 and <10.9 × 10^9^/l					1
≥ 10.9 and <14.0 × 10^9^/l					2
≥ 14.0 × 10^9^/l					3
Leukocytosis shift	1				
Polymorphonuclear leukocytes					
70–84 %		1			
≥ 75 %			1		
≥ 85 %		2			
≥ 62 % and < 75 %					2
≥ 75 % and < 83 %					3
≥ 83 %					4
CRP (C-reactive protein) concentration					
10–49 mg/l		1			
≥ 50 mg/l		2			
Symptoms <24 h and CRP (C-reactive protein) concentration					
≥ 4 and <11 mg/l					2
≥ 11 and <25 mg/l					3
≥ 25 and <83 mg/l					5
≥ 83 mg/l					1
Symptoms >24 h and CRP (C-reactive protein) concentration					
≥ 12 and <53 mg/l					2
≥ 53 and <152 mg/l					2
≥ 152 mg/l					1
Coughing/hopping/percussion pain			2		
Gender					
Male				1	
Female				0.5	
Age					
< 40 years				1	
≥ 40 years				0.5	
Duration of symptoms					
< 48 h				1	
> 48 h				0.5	
Negative urinalysis				1	
Total score	10	12	10	16.5	23

^a^Alvarado score: sum 0–4 = not likely appendicitis, sum 5–6 = equivocal, sum 7–8 = probably appendicitis, sum 9–10 = highly likely appendicitis

^b^Acute appendicitis response score (AIR): sum 0–4 = low probability, sum 5–8 = indeterminate group, sum 9–12 = high probability [161]

^c^Pediatric appendicitis score (PAS): ≥6 = appendicitis, ≤5 = observe

^d^Raja Isteri Pengiran Anak Saleha Appendicitis (RIPASA) score

^e^Adult Appendicitis Score (AAS): low risk (0–10 points), intermediate risk (11–15 points), high risk (≥16 points)

^f^ *right iliac fossa*

More recently, attempts have been made to incorporate imaging findings into diagnostic scoring systems. Atema et al.[9] described a scoring system that successfully distinguished complicated from uncomplicated acute appendicitis, reporting a negative predictive value of 94.7 % (in correctly identifying patients with uncomplicated disease). A diagnostic scoring system that incorporates imaging to the primary clinical diagnosis of acute appendicitis has not yet been developed [10].

The Alvarado score is the most extensively studied score (though this statement is biased by time; the Alvarado score has been around much longer than some of the newer scores, e.g. the AAS). Its validity has been summarised in a recent meta-analysis [11] including 5960 patients in 29 studies. According to Ohle et al., the score’s performance is dependent on the cut-off value: a clinical cut-off score of less than five can be applied to 'rule out' appendicitis with a sensitivity of 99 % (95 % CI 97 – 99 %) and a specificity of 43 % (36 – 51 %), while a cut-off score of less than seven results in a sensitivity of 82 % (76 – 86 %) and a specificity of 81 % (76–85 %), suggesting it is not sufficiently accurate to rule in or rule out surgery. Individual validation studies occasionally reported lower sensitivity, questioning the ability of the Alvarado score to reliably exclude appendicitis with a cut-off score of less than five [12, 13]. However, these concerns are not supported by the pooled meta-analysis of those data [11].

The Appendicitis Inflammatory Response (AIR) score has been proposed in 2008 by Andersson [6] and is based on eight variables, including C-reactive protein (CRP). The AIR score showed a significant better discriminating capacity when compared with the Alvarado score, with a ROC area of 0.97 vs. 0.92 for advanced appendicitis (*p* = 0.0027) and 0.93 vs. 0.88 for all appendicitis (*p* = 0.0007). According to the score, two cut-off points were identified to obtain three diagnostic test zones: a score <4 (low probability) has a high sensitivity (0.96) for appendicitis and can be used to rule out appendicitis; a score between five and eight identifies the intermediate probability patients, that require observation and eventual further investigations; a score >8 (high probability) has a high specificity (0.99) for appendicitis and can be used to rule in appendicitis. The AIR score has been also externally validated (ROC AIR 0.96 vs. Alvarado 0.82 *p* < 0.001) [14], especially in the high-risk patients, where a higher specificity and positive predictive value than the Alvarado score (97 vs. 76 % *p* < 0.05 and 88 vs. 65 % *p* < 0.05, respectively) has been reported [15]. The AIR score has demonstrated to be useful in guiding decision-making to reduce admissions, optimize utility of diagnostic imaging and prevent negative explorations [16].

Diagnostic scoring systems may perform differently in adult and paediatric patients. In fact, at a practical level, several of the predictor variables may be difficult to apply (e.g. asking an infant to describe migratory pain). The definition of a paediatric patient was not standardised among the studies, or clearly defined in the meta-analysis. Another systematic review compared the Alvarado score with the Paediatric Appendicitis Score, favouring the former [17].

The various derivation and validation studies investigating the different diagnostic scoring systems are troubled by various methodological weaknesses. Firstly, there is often inadequate definition of predictor variables, absence of reproducibility testing of predictor variables [18], lack of blinding and insufficient power [19]. Secondly, with regards to the participants, these studies often only include patients who an appendectomy was subsequently performed and for this reason potentially under-report false negatives. Such studies are questionable as the score is meant to be used on patients with suspicion of appendicitis, before all other diagnostic workup or selection. Thirdly, there is great variability in the study populations’ level of appendicitis (ranging between approximately 10 – 80 %); studies with a high rate of disease should demonstrate a higher specificity in their diagnostic scoring system. Regrettably, due to these multiple factors, there is a great deal of heterogeneity among the diagnostic studies used to derive and validate the diagnostic scoring systems described. This heterogeneity, differences in treatment systems, and the fundamental demographic differences in treatment cohorts confound the direct applicability of these clinical studies in other practices.

No data are available to evaluate the ability of the published diagnostic scoring systems to improve clinical outcomes (e.g. length of hospital stay, perforation rate, negative appendectomy rate).

No cost analysis of diagnostic scoring system for the clinical diagnosis of acute appendicitis was identified.

The sensitivity and specificity of the diagnostic scoring systems are inversely related. At the expense of specificity, scoring systems may be given sufficiently sensitive cut-off scores to exclude disease (e.g. Alvarado score < 5). However, none of the current diagnostic scoring systems can reach enough specificity to identify with absolute certainty which patients warrant an appendectomy.

**Statement 1.1 The Alvarado score (with cut-off score < 5) is sufficiently sensitive to exclude acute appendicitis. [EL 1, GoR A].**

**Statement 1.2 The Alvarado score is not sufficiently specific in diagnosing acute appendicitis [EL 1, GoR A].**

**Statement 1.3 An ideal (high sensitivity and specificity), clinically applicable, diagnostic scoring system/clinical rule remains outstanding. This remains an area for future research. [EL 1, GoR B]**

#### What is the value of clinical and laboratory findings in patients with suspected appendicitis?

The decision to do additional imaging of a patient with suspected appendicitis is based mainly on the complaints of the patient combined with findings at physical examination. The clinical presentation is, however, seldom typical and diagnostic errors are common. A thorough clinical examination is often stressed as an essential part of diagnosis, with laboratory examinations as an adjunct to the gathered clinical information. The review by Andersson [20] shows that each element of the history and of clinical and laboratory examinations is of weak discriminatory and predictive capacity. However, clinical diagnosis is a synthesis of information obtained from all these sources, and a high discriminatory and predictive power can be achieved by an accurate understanding of the relative importance of variables in combination. When the values of two or more inflammatory variables found in laboratory are normal, appendicitis is unlikely. Conversely, appendicitis is very likely when the values of two or more inflammatory variables are increased [21]. Laboratory tests of the inflammatory response and the clinical descriptors of peritoneal irritation and migration of pain are the strongest discriminators and should be included in the diagnostic assessment of patients with suspected appendicitis.

### Role of imaging

*What is the optimum pathway for imaging in patients with suspected acute appendicitis? Routine vs. selective imaging? CT or US or both? In what order?(Speaker in Jerusalem CC Dr. M. Sugrue)*

Diagnosis of AA is made by clinical history and physical examination the typical symptoms and laboratory signs may be absent in 20–33 % of patients and, when they are present, can be similar to other conditions, especially in early stage [22, 23] and the diagnosis can be particularly difficult in children, elderly patients, pregnant and childbearing age women.

Although several previous studies have shown discriminant factors in the differential diagnosis of AA and pelvic inflammatory disease (PID) in childbearing age women [24–29], imaging techniques such as US, CT or MRI may be required to reduce the negative appendectomy rate, with a low level of evidence currently available [30, 31]. Occasionally there is a role for diagnostic laparoscopy particularly in younger female patients [32].

In patients older than age 50 years diverticulosis is extremely common in the USA and Europe (about 8.5 % of the population) [33]. Right-sided diverticula occur more often in younger patients than do left-sided diverticula and because patients are young and present with right lower quadrant pain, they are often thought to suffer from acute appendicitis; it is difficult to differentiate solitary caecal diverticulitis from acute appendicitis. More than 70 % of patients with caecal diverticulitis were operated on with a preoperative diagnosis of acute appendicitis. In addition, selective focused imaging can be used for increasing the positive appendectomy rate imaging with aim to aid in diagnosing alternative diseases, who may not need surgery (e.g. omental infarction, solitary caecal diverticulum and torsion of appendix epilplocae). Nevertheless, delay in diagnosis later than 24 h increases risk of perforation, [34].

When recommending the choice of the imaging strategy, the patients’ age and the potential radiation exposure are important. Although a careful balance of risk-benefit ratio is needed, particularly in young patients and women of childbearing age, routine use of CT scan has been demonstrated to be associated with lower negative appendectomy rates [35]. Furthermore, there is increasing evidence that spontaneous resolution of AA is common and that imaging can lead to increased detection of benign forms of the condition [36].

In view of the increased use of CT in children and concerns regarding radiation based imaging, the National Cancer Institute and the American Paediatric Surgical Association recommend use of non-radiation based imaging such as US where possible [37]. Currently, over 50 % of children undergoing appendectomy in North America have radiation based imaging [38]. This rate is too high [39] and a tailored approach based on risk is sensible, especially in children. Universal imaging of patients with CT, apart from consuming resources, is not without health risks. It has been estimated that the benefit of universal imaging in avoiding 12 unnecessary appendectomies could result in one additional cancer death [40].

In pregnant women with suspected appendicitis a positive US requires no further confirmatory test. However, in case of appendix non-visualization on US, MRI is the recommended imaging exam, since it yields a high diagnostic rate and accuracy [41–43].

In settings having availability of such resource, MRI can also be considered for pediatric appendicitis imaging being a non-radiative imaging modality potentially valuable in the setting of negative ultrasound.

Imaging is key in optimizing outcomes in appendicitis, not only as an aid in early diagnosis, but potentially reducing negative appendectomy rates. Combining appropriate imaging with history, physical examination and laboratory tests are crucial to this [8, 19, 44–49]. With use of novel scoring systems combining clinical and imaging features, 95 % of the patients deemed to have uncomplicated appendicitis were correctly identified as such [9]. Soreide in a recent PubMed search under the term appendicitis found over 20,000 articles, but few randomized trials, especially in imaging, have been undertaken with resultant variable level of evidence [50]. Wide variation in rates of imaging as low as a CT rate of 12 % in the UK, to 95 % in the US suggests a need for practice guidelines [51]. Only 25 % of Australian patients undergo imaging [52].

The surgeon has the responsibility of managing each case in the best way considering three possibilities: hospital discharge, admission for observation, surgical treatment. Estimating pre-image likelihood of appendicitis is important in tailoring management: low-risk patients could be discharged with appropriate safety netting, whereas high-risk patients are likely to require early senior review with a focus on timely surgical intervention rather than diagnostic imaging [16]. Using scoring systems to guide imaging can be helpful [49, 53].

Low risk patients being admitted to hospital and considered for surgery could have appendicitis ruled in or out by abdominal CT. A negative CT would generally allow the discharge of the patient with appropriate short outpatient-department follow-up [16].

Intermediate-risk classification identifies patients likely to benefit from observation and systematic diagnostic imaging. In the intermediate risk group an abdominal ultrasound would be the first line in imaging. A positive ultrasound would lead to appendectomy and a negative test to either CT or further clinical observation. A conditional CT strategy, where CT is performed after a negative US, will reduce number of CTs by 50 % and will correctly identify as many patients with appendicitis as an immediate CT strategy. However, conditional CT imaging results in more false positives [9, 54]. Overall sensitivity and specificity of US and CT is 58–76, 95 and 99, 84 % respectively [9, 55]. Performing serial US may improve accuracy and reduce the number of CT performed [56].

High-risk scoring patients may not require imaging in certain settings, nonetheless US or CT before surgery is routinely performed in western countries in such patients [16].

Standard reporting templates for ultrasound may enhance accuracy [40]. To optimize sensitivity and specificity three step sequential positioning or graded compression bedside may be beneficial [55], as opposed to radiology department. US lacks Level 1/2 evidence to support its use [57], The routine use of IV contrast to enhance the accuracy of CT is not clear [58], nor is the role of dose reduction techniques.

Findings suggestive of appendicitis include a thickened wall, a non-compressible lumen, diameter greater than 6 mm, absence of gas in the lumen, appendicoliths, hyper-echogenic periappendicular fat, fluid collection consistent with an abscess, local dilation and hypoperistalsis, free fluid and lymphadenopathy [40]. The most sensitive sign seems to be a non-compressible appendix that exceeds 6 mm in diameter (up to 98 % sensitive), although some centres use 7 mm to improve their specificity [59]. As described earlier, ultrasound is inferior to CT in sensitivity and its negative predictive value for appendicitis and may not be as useful for excluding appendicitis [60]. This is particularly true if the appendix was never visualized. False negatives are also more likely in patients with a ruptured appendix. The potential adverse effect of high BMI on US accuracy is surprisingly not clear [61].

MRI is comparable to US with conditional use of CT in identifying perforated appendicitis. However, both strategies incorrectly classify up to half of all patients with perforated appendicitis as having simple appendicitis [62]. Scoring systems will enhance the ability to categorize whether appendicitis is simple or complex, showing that imaging is not a replacement for clinical examination. Finally, imaging may be undertaken by non-radiologists outside the radiology departments with variable results [63].

#### USA vs. EU perspective on appendicitis diagnosis

AA is rarely diagnosed by history/physical examination in the United States (USA). Unfortunately most of these patients in the USA are seen by emergency physicians and tests are ordered before the surgeon is called. In adults, it is rare to not obtain a CT scan unless a thin male (also rare in the USA). In children, an ultrasound is nearly always done. In the USA, logistics and legal concerns unfortunately impact our decision-making.

Despite the EU and the USA having similar access to health care, health technology and standards, they are very different healthcare systems with some inherent differences in the management strategies for appendicitis. One aspect that highlights this is the pre-operative imaging strategy for diagnosis. In the EU, only around 12.9 % of patients undergo pre-operative CT imaging [51]; which is typically reserved for elderly patients who might have cancer, atypical or delayed presentations or those who have suspected appendicular masses or abscesses. Young males with typical histories and examination findings would go straight to theatre without any imaging. Females would get an abdominal and pelvic ultrasound and laparoscopy if uncertainty exists. Perhaps as a consequence of this strategy, the rate of negative appendectomy in the UK is around 20 % [64]; this is in contrast to the USA. For instance, analysis of 3540 appendectomies form the Surgical Care and Outcomes Assessment Programme (SCOAP) in Washington State demonstrates that 86 % of patients underwent pre-operative imaging, 91 % of whom underwent CT [65]. In addition, in the UK, appendectomy is widely regarded as a training operation that most registrars would perform independently. From 2867 appendectomies in the recent UK audit, 87 % were performed by residents, and 72 % were performed unsupervised [66]. Laparoscopic appendectomy is performed, especially in high volume units, during the daytime and when a consultant is present in theatre, but overall 33.7 % of cases are performed as open procedures [51].

**Statement 2.1 In patients with suspected appendicitis a tailored individualised approach is recommended, depending on disease probability, sex and age of the patient (EL 2 GoR B) Statement 2.2 Imaging should be linked to Risk Stratification such as AIR or Alvarado score. (EL2, GoR B)**

**Statement 2.3 Low risk patients being admitted to hospital and not clinically improving or re-assessed score could have appendicitis rule-in or out by abdominal CT. (EL 2, GoR B)**

**Statement 2.4 Intermediate-risk classification identifies patients likely to benefit from observation and systematic diagnostic imaging. (EL 2, GoR B)**

**Statement 2.5 High-risk patients (younger than 60 years-old) may not require pre-operative imaging. (EL 2, GoR B)**

**Statement 2.6 US Standard reporting templates forultrasound and US three step sequential positioningmay enhance over accuracy. (EL 3, GoR B)**

**Statement 2.7 MRI is recommended in pregnant patients with suspected appendicitis, if this resource is available. (EL 2, GoR B)**

### Non-operative treatment for uncomplicated appendicitis

*What is the natural history of appendicitis? Can appendicitis resolve without treatment? How common is it?(Speaker in Jerusalem CC Dr. F. Catena)*

The analysis of the epidemiologic and clinical studies that elucidate the natural history of appendicitis performed by Andersson in 2007 showed that not all patients with uncomplicated appendicitis will progress to perforation and that spontaneous resolution may be a common event [36]. Also the recent review published in The Lancet investigated the natural history of appendicitis and distinguished between normal appendix, uncomplicated appendicitis and complicated appendicitis, according to their macroscopic and microscopic appearance and clinical relevance. Actually, if this is related to the natural history of appendicitis or not is still unknown, but according to the authors these may be two distinct forms of appendicitis: the first one is a mild simple appendicitis that responds to antibiotics or could be even self-limiting, whereas the other often seems to perforate before the patient reaches the hospital. Although the mortality rate is low, postoperative complications are common in case of complicated disease [67].

In order to elucidate the role of non-operative treatment of uncomplicated appendicitis, in 2012 Varadhan et al. performed a meta-analysis including four randomized controlled trials with a total of 900 patients (470 antibiotic treatment, 430 appendectomy): the antibiotic treatment was associated with a 63 % success rate at 1 year and a lower complication rate with a relative risk reduction of 31 % if compared with appendectomy (RR 0.69, I2 = 0 %, *P* = 0.004). Moreover, this risk reduction was found to be more relevant (39 %, RR 0.61, I2 = 0 %, *P* = 0.02), if the studies with crossover of patients between the antibiotic and surgical treatment were excluded. The analysis did not find significant differences for treatment efficacy, length of stay or risk of developing complicated appendicitis [2].

The observational NOTA (Non Operative Treatment for Acute Appendicitis) study treated 159 patients with suspected appendicitis with antibiotics [mean AIR (Appendicitis Inflammatory Response) score = 4.9 and mean Alvarado score = 6.2 (range 3–9) [68]] with a 2-year follow-up. The mean length of stay of those patients was 0.4 days and mean sick leave period was 5.8 days. The short-term (7 days) failure rate was 11.9 %. Of 22 patients with a long-term recurrence (13.8 %), 14 were successfully treated nonoperatively [69].

Recently, the RCT by Svensson et al. included 50 paediatric patients (24 antibiotic treatment, 26 appendectomy) with 92 % of success rate in the non-operative group. However, an 8 % short-term failure (two patients, one complicated appendicitis and one mesenteric lymphadenitis) and 38 % long-term (12 months) failure were reported in the non-operative group (one acute appendicitis, six patients with recurrent abdominal pain but no histopathological evidence of appendicitis and one for parental wish) [70].

The APPAC (Antibiotic Therapy vs Appendectomy for Treatment of Uncomplicated Acute Appendicitis) trial, published in JAMA in 2015, enrolled 350 patients with uncomplicated appendicitis confirmed by CT-scanning (257 antibiotic therapy, 273 appendectomy). The 1-year recurrence rate and appendectomy in the antibiotic group was reported as 27 %. The intention-to-treat analysis yielded a difference in treatment efficacy between groups of −27.0 %(95%CI, −31.6 % to ∞) (*P* = .89). The authors concluded that the antibiotic treatment did not meet the pre-specified criterion for non-inferiority compared with appendectomy [71].

In the recent review published in the New Engl J Med by Flum it is stated that appendectomy should be considered the first-line therapy in uncomplicated appendicitis and recommended to the patient. In the patients with equivocal clinical picture, or equivocal imaging, or in those who have strong preferences for avoiding an operation or with major comorbid medical problems it is reasonable to treat with antibiotics first [72].

However, an interesting still not well-studied topic is the role of spontaneous resolution of uncomplicated appendicitis. In fact, the effect of the antibiotic treatment could be biased due to spontaneous healing as a result of the expectant management [47].

**Statement 3.1: Antibiotic therapy can be successful in selected patients with uncomplicated appendicitis who wish to avoid surgery and accept the risk up to 38 % recurrence. (EL 1, GoR A)**

**Statement 3.2: Current evidence supports initial intravenous antibiotics with subsequent conversion to oral antibiotics. (EL2, GoR B)**

**Statement 3.3: In patients with normal investigations and symptoms unlikely to be appendicitis but which do not settle:**

- Cross-sectional imaging is recommended before surgery
  - Laparoscopy is the surgical approach of choice
  - There is inadequate evidence to recommend a routine approach at present (EL2 GoR)

### Timing of appendectomy and in-hospital delay

*Does in-hospital delay increase the rate of complication or perforation? Is it safe to delay appendectomy? Timing of appendectomy. (Speaker in Jerusalem CC Dr. M.D. Kelly)*

The management of most intra-abdominal acute surgical conditions has evolved significantly over time and many are now managed without emergency operation. Since the 1880s, when Fitz and McBurney described emergency appendectomy, it has been the standard of care for suspected appendicitis. This is based on the traditional model of appendicitis where initial obstruction causes inflammation and infection, and delay to operation allows increasing tension in the wall with ischemia, necrosis and perforation. This pathophysiology probably does not fit with all cases of appendicitis, as discussed below, and emergency operation is not always needed.

Delay to appendectomy may be needed for various reasons, including a trial of conservative treatment with antibiotics, diagnostic tests to confirm the clinical diagnosis or to allow safe service provision and effective use of resources as not all hospitals are staffed or set up for 24 h operating room availability. Whatever the cause for delay, the real issue is if it will lead to more complications: there are numerous studies looking at the question of in-hospital delay and indirect evidence can be obtained from randomised trials of antibiotics versus surgery, however controversy persists.

A recent publication had a 27 % negative appendectomy rate and the authors justify their low threshold to operate by stating that it avoids perforation [73]. Others disagree and found that delaying surgical intervention did not put the patient at risk and may have actually improved patient outcomes [74]. The current diversity in practice appears to be caused by lack of high-level evidence although this is beginning to change. It should be noted that the danger of perforation is possibly overstated and that negative exploration is not benign [36].

Conservative management decreases the number of negative explorations and saves a number of patients with resolving appendicitis from an unnecessary operation. Andersson has shown that this leads to a high proportion of perforations among the operated patients but the number of perforations is not increased. The perforation rate, therefore, should not be used as a quality measure of the management of patients with suspected appendicitis [36]. He also notes that the increasing proportion of perforations over time is explained by an increase in the number of perforations according to the traditional model and mainly by selection due to resolution of non-perforated appendicitis according to the alternative model. According to the second model, only a few perforations can be prevented by a speedy operation after the patients have arrived at the hospital. Neither of these models can be proved, but the second model is more consistent with the available data [36].

Similarly, others have found that the trends for non-perforating and perforating appendicitis radically differ and it is unlikely that perforated appendicitis is simply the progression of appendicitis resulting from delayed treatment [75].

There are numerous retrospective single institution reviews with contradictory results.

Teixeira et al. found only increased rates of surgical site infection. They studied 4529 patients who were admitted with appendicitis over 8 years and 4108 (91 %) patients underwent appendectomy with perforation found in 942 (23 %). There were three independent predictors of perforation: age > 55 years, WBC count >16,000 and female sex, but delay to appendectomy was not associated with higher perforation rate [76]. However, Ditillo et al. found that increased patient and hospital intervals to operation were associated with advanced pathology, although patient delay was more significant. The risk of developing advanced pathology increased with time and it was associated with longer length of hospital stay and antibiotic treatment as well as postoperative complications [77].

In a large retrospective cohort study of 32,782 patients who underwent appendectomy for acute appendicitis (available through the American College of Surgeons National Quality Improvement Program), 75 % of patients underwent operation within 6 h, 15 % between 6 and 12 h and 10 % of patients experienced a delay of more than 12 h (mean 26.07 h (SD 132.62)). The patient characteristics were similar in all three groups. No clinically significant difference was found in outcome measures, including overall morbidity and serious morbidity or mortality. The authors concluded that the results did not change when disease severity was excluded from the model suggesting that there is no relationship between time from surgical admission and negative outcomes after appendectomy [78].

Busch et al. reported a prospective multicentre observational study on whether in-hospital delay negatively influences outcome after appendectomy. In-hospital delay of more than 12 h, age over 65 years, time of admission during regular hours, and the presence of co-morbidity are all independent risk factors for perforation. Perforation was associated with a higher re-intervention rate and increased hospital length of stay. They concluded that in elderly patients with co-morbidity and suspected appendicitis, a delay of surgery of more than 12 h should be avoided [79].

As can be seen, the evidence is conflicting but recently higher level evidence has become available in the study by Bhangu et al. This was a prospective, multicentre cohort study of 2510 patients with acute appendicitis, of whom 812 (32.4 %) had complex findings. They found that timing of operation was not related to risk of complex appendicitis. At 12–24 h, the odds ratio (OR) was 0.98 (*P* = 0.869), 24–48 h OR 0.88 (*P* = 0.329) and 48+ hours OR 0.82 (*P* = 0.317). After 48 h, the risk of surgical site infection and 30-day adverse events both increased [adjusted ORs 2.24 (*P* = 0.039) and 1.71 (*P* = 0.024), respectively]. They also did a meta-analysis of 11 nonrandomized studies (8858 patients) which showed that a delay of 12 to 24 h after admission did not increase the risk of complex appendicitis (OR 0.97, *P* = 0.750) [34].

In some jurisdictions, after hours surgery (especially night time surgery) is restricted to life or limb-threatening conditions as not all hospitals are staffed or equipped for safe 24-h operating room availability. In addition, especially in state funded health systems, where all expenditure has to be based on evidence, it is hard to justify after hours surgery for uncomplicated appendicitis.

There are now many randomised studies of initial antibiotic treatment for appendicitis. While not designed to look at delay to operation, they give indirect evidence of its safety in patients with uncomplicated appendicitis [2, 71, 80].

In summary, in the absence of level 1 evidence, the question of whether in-hospital delay is safe and not associated with more perforations cannot be answered with certainty. What can be said is that in most cases of uncomplicated appendicitis emergency operation is not necessary and a short delay of up to 12–24 h is not likely to be associated with a poorer outcome. However, delays should be minimised wherever possible to relieve pain, to enable quicker recovery and decrease costs.

**Statement 4.1 Short, in-hospital surgical delay up to 12/24 h is safe in uncomplicated acute appendicitis and does not increase complications and/or perforation rate. (EL 2, GoR B)]**

**Statement 4.2 Surgery for uncomplicated appendicitis can be planned for next available list minimizing delay wherever possible (patient comfort etc.). (EL 2, GoR B)**

### Surgical treatment

- *Open or Laparoscopic?*
- *Lavage or Aspiration of pus?*
- *Mesoappendix dissection: endoclip, endoloop, electrocoagulation, Harmonic Scalpel or LigaSure?*
- *Stump Closure: Stapler or endoloop? Ligation or invagination of the stump?*
- *Drains?*
- *Primary or secondary closure of the wound?*

*(Speaker in Jerusalem CC Dr. S. Di Saverio)*

The most recent meta-analysis reported that the laparoscopic approach of appendicitis is often associated with longer operative times and higher operative costs, but it leads to less postoperative pain, shorter length of stay (LOS) and earlier return to work and physical activity [81] therefore lowering overall hospital and social costs [82], improved cosmesis, significantly fewer complications in terms of wound infection. A trend towards higher incidence of intra-abdominal infection (IAA) and organ space collections was seen [83], although this effect seems decreased or even inverted in the last decade [84] and in more recent randomised controlled trials (RCTs), being probably related to surgical expertise [85].

According to Sauerland et al., wound infections are less likely after laparoscopic appendectomy (LA) than after open appendectomy (OA) (OR 0.43; CI 0.34 to 0.54), pain on day 1 after surgery is reduced after LA by 8 mm (CI 5 to 11 mm) on a 100 mm visual analogue scale, hospital stay was shortened by 1.1 day (CI 0.7 to 1.5), return to normal activity, work, and sport occurred earlier after LA than after OA. However, as we said, the incidence of IAA is increased (OR 1.87; CI 1.19 to 2.93). In addition, the operation time is 10 min (CI 6 to 15) longer and more expensive. Seven studies on children were included, but the results do not seem to be much different when compared to adults. Diagnostic laparoscopy reduces the risk of a negative appendectomy, but this effect was stronger in fertile women (RR 0.20; CI 0.11 to 0.34) as compared to unselected adults. The authors conclude the in those clinical settings where surgical expertise and equipment are available and affordable, diagnostic laparoscopy and LA (either in combination or separately) seem to have numerous advantages over OA [83].

The overview by Jaschinski et al. included nine systematic reviews. The duration of surgery pooled by eight reviews was 7.6 to 18.3 min shorter using the open approach and the risk of abdominal abscesses was higher for laparoscopic surgery in half of six meta-analyses. The laparoscopic approach shortened hospital stay from 0.16 to 1.13 days in seven out of eight meta-analyses, pain scores on the first postoperative day were lower after LA in two out of three reviews and the occurrence of wound infections pooled by all reviews was lower after LA. One review showed no difference in mortality [86].

Although LA is extremely useful especially as a diagnostic tool in fertile women, in can be used also in male patients, even if advantages over OA in this group are not clearly demonstrated [87].

Recent database studies on more than 250,000 patients aged > 65 years entail improved clinical outcomes for laparoscopic appendectomy compared with OA [88] in terms of length of stay (LOS), mortality and overall morbidity. Patients older than 65 years, patients with comorbidities [89] and with complicated appendicitis [90] seem to benefit more from the laparoscopic approach, particularly in terms of hospital costs and reduced LOS but also for decreased postoperative mortality and overall morbidity [91].

A meta-analysis of prospective and retrospective comparative series evidences superiority of LA vs. OA also in obese (BMI >30) patients [92]. Dasari et al. reported the same encouraging results also in a recent Systematic Review [93].

Despite evidence which considers LA safe in pregnancy [94], advantages are minor (less pain, less infections, less early deliveries) if compared to the risk of fetal loss; more recent data from EL 2 reviews of comparative studies (599 LA vs. 2816 OA) show an increased fetal loss for LA, without significant advantages [95]; a database study on 859 pregnant women with appendicitis confirms a better outcome for those treated surgically vs. non-operative management, while it did find no difference in maternal complications between LA and OA [96]. While fetal events are unknown, LA in pregnant patients demonstrated shorter OR times, LOS, and reduced complications and were performed more frequently over time. Even in perforated cases, laparoscopy appears safe in pregnant patients [97]. In conclusion, there is no strong current evidence as to the preferred modality of appendectomy, open or laparoscopic, during pregnancy from the prospect of foetal or maternal safety. However, low grade evidence shows that laparoscopic appendectomy during pregnancy might be associated with higher rates of foetal loss [98]. The literature does not clearly define the balance between advantages and disadvantages in this particular setting and the choice of the approach should be taken by the attending surgeon after a thorough discussion with the patient, balancing the advantages of laparoscopy vs. the theoretical risk of fetal loss and making clear the current lack of literature defining balance between advantages and disadvantages of laparoscopic appendectomy in pregnancy.

A recent systematic review including more than 100.000 appendectomies in children found that laparoscopic appendectomy in uncomplicated acute appendicitis is associated with a reduced hospital stay (weighted mean difference 0–1.18; 95 % CI0 − 1.61 to −0.74; *P* < 0.05), but broad equivalence in postoperative morbidity when compared with the conventional approach. On the other hand, in cases of complicated acute appendicitis, although the overall morbidity is reduced (pooled odds ratio [POR] = 0.53; *P* < 0.05), wound infections (POR = 0.42; *P* < 0.05), length of hospital stay (WMD = −0.67; *P* < 0.05), and bowel obstruction episodes (POR = 0.8; *P* < 0.05), in the laparoscopic group the risk of intra-abdominal abscess is increased [99].

Complicated appendicitis can be approached laparoscopically by experienced surgeons [100], with significant advantages, including lower overall complications, readmission rate, small bowel obstruction rate, infections of the surgical site (minor advantage following Clavien's criteria) and faster recovery [89, 101, 102]. Regarding the costs, LA for complicated appendicitis can be performed with low cost equipment, allowing significantly lower overall costs (operative plus LOS) compared to open surgery [103].

**Statement 5.1.1:**

**Laparoscopic appendectomy should represent the first choice where laparoscopic equipment and skills are available, since it offers clear advantages in terms of less pain, lower incidence of SSI, decreased LOS, earlier return to work and overall costs. (EL 1, GoR A)**

**Statement 5.1.2:**

**Laparoscopy offers clear advantages and should be preferred in obese patients, older patients and patients with comorbidities. (EL 2, GoR B)**

**Statement 5.1.3:**

**Laparoscopy is feasible and safe in young male patients although no clear advantages can be demonstrated in such patients. (EL 2, GoR B)**

**Statement 5.1.4:**

**Laparoscopy should not be considered as a first choice over open appendectomy in pregnant patients. (EL 1, GoR B)**

**Statement 5.1.5:**

**No major benefits have also been observed in laparoscopic appendectomy in children, but it reduces hospital stay and overall morbidity. (EL 1, GoR A)**

**Statement 5.1.6:**

**In experienced hands, laparoscopy is more beneficial and cost-effective than open surgery for complicated appendicitis. (EL 3, GoR B)**

Peritoneal lavage and aspiration have been suggested by a low-powered study to be detrimental, but these conclusions are based on low-volume lavage and small numbers [104]; a definitive conclusion cannot be drawn, even though a LE 2 study in children [105] has not demonstrated advantages in terms of intra-abdominal abscesses (IAA) of >500 ml, although >6–8lt are needed to significantly lower the bacterial load [106].

Peritoneal irrigation is a practice traditionally used in case of localized or diffuse peritonitis and considered beneficial. However, either in the past decades for open appendectomy or in the latest years for laparoscopic appendectomy, many others argued the efficacy of irrigation for cleansing purposes. The most recent studies, retrospective [104] or RCTs, in laparoscopic or open appendectomy [107], did not show any advantages in favour of intraoperative irrigation for prevention of postoperative IAA. Instead, irrigation usually adds some extra-time to the overall duration of surgery [105]. Nonetheless, a non-significant trend to leave a drain when irrigation is not used can be noticed (52 % in the group of suction only vs. 40 % in the irrigation group). Furthermore, practice patterns may vary widely with regard to the amount and extent of irrigation and probably the common sense would suggest to avoid copious irrigation before achieving a careful suction first from every quadrant having purulent collections and to wash using small amounts of saline and repeated suction in order to avoid diffuse spreading of the infected matter into the remaining abdominal cavity, without forgetting to suck out as much as possible of the lavage fluid [108].

**Statement 5.2:**

**Peritoneal irrigation does not have any advantages over suction alone in complicated appendicitis. (EL2, GoR B)**

Simplified and cost effective techniques for LA have been described [109]. They use either two endoloops, securing the blood supply, or a small number of endoclips, appearing to be really useful in case of mobile cecum avoiding the need of an additional port. In addition, potential hazards of diathermy are avoided, the appendicular artery can be ligated under direct vision, and smoke is not created [110]. With clips, anonabsorbable foreign body is left in the peritoneal cavity and may slip or become detached. Moreover, it requires more experience especially in case of inflamed appendix with the risk of bleeding [111–113].

In case of inflamed and oedematous mesoappendix it has been suggested the use of LigaSure™, especially in case of gangrenous tissue [112, 113]. No significant hospital stay and complication rates were found between endoclip and LigaSure™. On the other hand, significant differences are present in surgical time and conversion to open rate [111]. Despite the potential advantages, Ligasure™ represents a high cost option and it may be logical using endoclip if the mesoappendix is not oedematous [111–113]. Diamantis et al. compared Ligasure™ and Harmonic Scalpel with monopolar electrocoagulation and bipolar coagulation: the first two caused more minimal thermal injury of the surrounding tissue than other techniques [114]. Between monopolar electrocoagulation, endoclip and Harmonic Scalpel no clinically significant differences were found in surgical time. All three methods gave acceptable complication rates. Because monopolar electrocoagulation requires no additional instruments, it may be the most cost-effective method for mesoappendix dissection in LA [115]. However, the need of evacuate of the smoke could affect the pneumoperitoneum [111].

**Statement 5.3.1:**

**There are no clinical differences in outcomes, LOS and complications rates between the different techniques described for mesentery dissection (monopolar electrocoagulation, bipolar energy, metal clips, endoloops, Ligasure, Harmonic Scalpel etc.). (EL3, GoR B)**

**Statement 5.3.2:**

**Monopolar electrocoagulation and bipolar energy are the most cost-effective techniques, even if more experience and technical skillsis required to avoid potential complications (e.g. bleeding) and thermal injuries. (EL3, GoR B)**

As for appendicular stump closure, stapler reduces operative time and superficial wound infections [116], but higher costs (6 to 12 fold) and no significant differences in IAA [117], suggest the preference of loop-closure. In perforated appendicitis the issue of using endoloops or stapler for appendicular stump closure needs further studies [118].

The stump closure may vary widely in practice and the associated costs can be significant. Whilst earlier studies initially reported advantages with routine use of endostaplers in terms of complication and operative times [116], more recent studies have repeatedly demonstrated no differences in intra- or post-operative complications incidence between either endostapler or endoloops stump closure [119]. Although operative times maybe longer (but it is probably biased by the learning curve) [120], the operative costs were invariably and significantly lower when endoloops are used [103, 121]. A metanalysis confirmed that use of endo-loop to secure the appendicular stump during LA takes longer than endo-GIA but it is associated with equal hospital stay, perioperative complication rate, and incidence of intra-abdominal abscess [122]. Endoloops were at least as safe and effective as endostapler also in paediatric population, without stump leaks nor differences in SSI and IAA in the group of non perforated appendicitis, whereas for perforated appendicitis, endoloops were perhaps safer than endostapler (IAA incidence 12.7 % vs. 50 %, OR 7.09) [123].

Many studies compared the simple ligation and the stump inversion and no significant differences were found [103, 124–127].

**Statement 5.4.1:**

**There are no clinical advantages in the use of endostapler over endoloops for stump closure for both adults and children. (EL 1, GoR A)**

**Statement 5.4.2:**

**Endoloops might be preferred for lowering the costs when appropriate skills/learning curve are available. (EL 3, GoR B)**

**Statement 5.4.3: There are no advantages of stump inversion over simple ligation, either in open or laparoscopic surgery. (EL 2, GoR B)**

Routine drainage has not proven its utility, with the exception of generalized peritonitis, and seems to cause more complications, LOS and transit recovery time [128], despite the widespread opinion that aspiration of the residual fluid after peritoneal lavage in the first 24 h postoperatively might lower the incidence of IAA in case of insufficient lavage [118].

The practice of leaving intra-abdominal drains is also widely used when complicated/perforated appendicitis is found. Mostly from paediatric experiences, it seems that the use of drainage and irrigation is associated with significantly longer operative times and LOS, without a decrease in post-operative infectious complications (instead a non-significant trend to more frequent wound infection and dehiscence, more IAA and longer postoperative ileus) [107].

Previous studies in children with perforated appendicitis have already reported a significantly lower incidence of SSI and IAA and better postoperative course in the group treated without peritoneal drainage [129].

This year, the meta-analyses by Cheng et al. included five trials involving 453 patients with complicated appendicitis who were randomised to the drainage group (*n* = 228) and the no drainage group (*n* = 225) after emergency open appendectomies and found no significant differences between the two groups in the rates of intra-peritoneal abscess or wound infection. The hospital stay was longer in the drainage group than in the no drainage group (MD 2.04 days; 95 % CI 1.46 to 2.62) (34.4 % increase of an 'average' hospital stay) [96].

**Statement 5.5.1:**

**Drains are not recommended in complicated appendicitis in paediatric patients. (EL3, GoR B)**

**Statement 5.5.2:**

**In adult patients, drain after appendectomy for perforated appendicitis and abscess/peritonitis should be used with judicious caution, given the absence of good evidence from the literature. Drains did not prove any efficacy in preventing intra-abdominal abscess and seem to be associated with delayed hospital discharge. (EL1, GoR A)**

In the most recent metanalysis investigating the advantages of delayed primary wound closure (DPC) vs. primary closure (PC) in contaminated abdominal operations DPC had a significantly longer length of stay than PC (1.6 days, 95 % CI: 1.41, 1.79). Two meta-analysis failed to prove the superiority of delayed primary skin closure in significantly reducing SSI (odds ratio 0.65; 95 % CI, 0.25–1.64; *P* = .36) [64] (risk ratio 0.89; 95 % CI: 0.46, 1.73) [130]. Similar result were achieved also in the paediatric population [131]. In addition, there is no evidence for any short-term or long-term advantage in peritoneal closure for non-obstetric operations [132].

**Statement 5.6:**

**Delayed primary skin closure does not seem beneficial for reducing the risk of SSI and increase LOS in open appendectomies with contaminated/dirty wounds. (EL1, GoR A)**

### Scoring systems for intra-operative grading of appendicitis and their clinical usefulness

*What are the histopathological criteria for appendicitis of clinical importance? Minor inflammatory changes, early appendicitis, catarrhal appendicitis. The criteria used will have an influence on the proportion of negative appendectomy, and also on evaluation of diagnostic performance. (Speaker in Jerusalem CC Dr. C. A. Gomes)*

The systematic review by Swank et al. reported the incidence of unexpected findings in the histopathological examination of the surgical specimen after appendectomy as 0.5 % of benign neoplasm, 0.2 % of malignant neoplasms, 0–19 % of parasitic infection, endometriosis in 0 % and granulomatosis in 0–11 % of cases. Most patients with malignant neoplasms, parasite infection and granulomatosis underwent additional investigation or treatment [133].

Apart from the unexpected findings, there is a lack of validated system for histological classification of acute appendicitis and controversies exist on this topic. The paper by Carr proposes basic and classical but practical findings about the histological diagnosis of acute appendicitis. The author assesses three important disease aspects: appendix gross appearance, microscopic findings and clinical significance. The most important concept in the diagnosis of acute appendicitis is the transmural inflammation. “Endoappendicitis” is a histological finding, but its clinical significance is not clear. The term “periappendicitis” refers to inflammation outside the appendix and its most common causes are gynaecological disorders like salpingitis and pelvic peritonitis [134].

The issue of the removal indication in case of “normal-looking” appendices is still under debate and there are conflicting studies showing the pros and cons of the appendectomy. According to the retrospective study by Grimes et al., including 203 appendectomies performed with normal histology, appendicular faecaliths may be a cause of right iliac fossa pain in the absence of obvious appendicular inflammation. In this study, the policy of routine removal of a normal-looking appendix at laparoscopy in the absence of any other obvious pathology appeared to be an effective treatment for recurrent symptoms in those cases with a faecalith [135]. The study by Van den Broek et al. concluded that it is safe to leave a normal looking appendix in place when a diagnostic laparoscopy for suspected appendicitis is performed, even if another diagnosis cannot be found at laparoscopy [136]. On the other hand, in the retrospective study by Phillips et al., almost one-third of apparently normal appendices being inflamed histologically. For this reason the authors would advocate the removal of a normal looking appendix in the absence of other explanatory pathology [137]. Recently, Lee et al. compared the postoperative complications after removal of an inflamed or non-inflamed appendix and found no difference between the two groups. The authors conclude that negative appendectomy should not be undertaken routinely during laparoscopy for right iliac fossa pain [138]. In the Multicentre Appendectomy Audit by Strong et al., 138 out of 496 specimens (27.8 %) judged as normal by the operating surgeon were found to be inflamed at the histopathological assessment [139].

In order to evaluate the appendix during diagnostic laparoscopy, in 2013 Hamminga et al. proposed the LAPP (Laparoscopic APPpendicitis) score (six criteria), with a single-centre prospective pilot study (134 patients), reporting high positive and negative predictive values, 99 and 100 %, respectively. However, the score still needs to be validated within a multicentre study [140].

In 2014 also the AAST proposed a system for grading severity of emergency general surgery diseases based on several criteria encompassing clinical, imaging, endoscopic, operative, and pathologic findings, for eight commonly encountered gastrointestinal conditions, including acute appendicitis, ranging from Grade I (mild) to Grade V (severe) [141].

In the recent multicentre cohort study by Strong et al. involving 3138 patients from five centres, the overall disagreement between the surgeon and the pathologist was reported in 12.5 % of cases (moderate reliability, k 0.571). In particular, 27.8 % of appendices assessed as normal by the surgeon revealed a pathology at histopathological assessment, while in 9.6 % of macroscopically appearing inflamed appendicitis revealed to be normal. Interestingly, the surgeon’s experience did not affect the disagreement rate. These findings suggest that surgeons' judgements of the intra-operative macroscopic appearance of the appendix is inaccurate and does not improve with seniority and therefore supports removal at the time of surgery [139]. Nonetheless, the clinical significance of these early and/or mild forms of microscopic appendicitis is still unclear at present.

The prospective study by Gomes et al. enrolled 186 patients with presumed acute appendicitis underwent appendectomy if diagnostic laparoscopy showed appendicitis or normal-looking appendix without any other intra-abdominal disease. The appendix was graded by the surgeon upon its visual appearance: grade 0 (normal looking), 1 (redness and oedema), 2 (fibrin), 3A (segmental necrosis), 3B (base necrosis), 4A (abscess), 4B (regional peritonitis), and 5 (diffuse peritonitis). This was then compared with a biochemical-histologic assessment of the removed appendix. The sensitivity, specificity, and accuracy of the laparoscopic grading system were 63, 83.3, and 80.1 %, respectively, and presented moderate concordance [k = 0.39 (95 % confidence interval, 0.23–0.55)]. The biochemical-histological diagnosis changed for 48 (25.8 %) patients who had been previously classified by surgeons during laparoscopy. Most incorrect grading occurred in grades 0 and 1 appendicitis [142]. The Gomes intraoperative grading score system is able to distinguish complicated appendicitis from uncomplicated cases has been externally validated [103] and may be useful for guiding postoperative management (e.g. use of antibiotics, antibiotic duration, LOS) and comparing therapeutic outcomes [143].

**Statement 6.1: The incidence of unexpected findings in appendectomy specimens is low but the intra-operative diagnosis alone is insufficient for identifying unexpected disease. From the current available evidence, routine histopathology is necessary. (EL 2, GoR B)**

**Statement 6.2: There is a lack of validated system for histological classification of acute appendicitis and controversies exist on this topic. (EL 4, GoR C)**

**Statement 6.3: Surgeon’s macroscopic judgement of early grades of acute appendicitis is inaccurate. (EL 2, GoR B)**

**Statement 6.4: If the appendix looks “normal” during surgery and no other disease is found in symptomatic patient, we recommend removal in any case. (EL 4, GoR C)**

**Statement 6.5: We recommend adoption of a grading system for acute appendicitis based on clinical, imaging and operative findings, which can allow identification of homogeneous groups of patients, determining optimal grade disease management and comparing therapeutic modalities. (EL 2, GoR B)**

### Non-surgical treatment for complicated appendicitis: abscess or phlegmone

*Role of percutaneous drainage and Interval Appendectomy or immediate surgery. (Speaker in Jerusalem CC Dr. M. De Moya)*

The study with highest level of evidence about the conservative treatment of complicated appendicitis with abscess or phlegmon is the meta-analysis by Simillis et al., published in 2010. It included 17 studies (16 nonrandomized retrospective and one non-randomized prospective) for a total of 1572 patients (847 treated with conservative treatment and 725 with appendectomy). Data revealed that conservative treatment was associated with significantly less overall complications (wound infections, abdominal/pelvic abscesses, ileus/bowel obstructions, and re-operations) if compared to immediate appendectomy. No significant difference was found in the duration of the first hospitalization, the overall hospital stay and the duration of intravenous antibiotics [144].

On the other hand, the recent randomized controlled trial by Mentula et al. compared the results from 60 patients with appendicular abscess treated either with immediate laparoscopic surgery (30 patients) or with conservative treatment (30 patients). The results showed that there was no difference in hospital stay between the two groups. In the laparoscopy group there were significantly fewer unplanned readmissions (3 % versus 27 %, *P* = 0.026), even if this group had 10 % risk for bowel resection and 13 % risk for incomplete appendectomy. The conservative group, instead, required more additional interventions (surgery or percutaneous drainage) (30 % versus 7 %, *P* = 0.042). Open surgery was required in three (10 %) patients in the laparoscopy group and in four (13 %) patients in the conservative group. The rate of uneventful recovery was 90 % in the laparoscopy group versus 50 % in the conservative group (*P* = 0.002). These data brought to the conclusion that several factors support the use of immediate surgery in patients with appendicular abscess [145]. However, it should be highlighted that laparoscopic appendectomy as first line approach, is a feasible and safe alternative to non-operative management +/− percutaneous drain only in presence of specific laparoscopic experience and advanced skills [146].

In the systematic review and meta-analysis by Andersson et al., including 61 studies (mainly retrospective studies, three randomized controlled trials), immediate surgery was associated with a higher morbidity if compared with conservative treatment (OR 3.3; CI: 1.9–5.6; *P* < 0.001), while the non-surgical treatment of appendicular abscess or phlegmon has been reported to succeed in over 90 % of patients, with an overall risk of recurrence of 7.4 % (CI: 3.7–11.1) and only 19.7 % of cases of abscess percutaneous drainage [3]. Other single-centre studies including complicated appendicitis reported higher rates of recurrence after non-surgical treatment of 14 % after 2 years [69], 27 % within 2 months [145], up to 38 % after 12 months [70]. In order to avoid this quite high chance of recurrence, some authors recommend routine elective interval appendectomy following the conservative management. However, this procedure is associated with morbidity in 12.4 % of patients (CI 0.3–24.5) [3]. The systematic review by Hall et al. included three retrospective studies for a total of 127 cases of non-surgical treatment of appendix mass in children: after successful non-operative treatment, the risk of recurrent appendicitis was found to be 20.5 % (95 % confidence interval [CI], 14.3 %–28.4 %). However, this means that 80 % of children may not need interval appendectomy. In addition, the results showed 0.9 % of carcinoid tumor (95 % CI, 0.5–1.8) and 3.4 % of complications after interval appendectomy (95 % CI, 2.2–5.1). Overall, the complications reported included wound infection, prolonged postoperative ileus, hematoma formation, and small bowel obstruction, but the incidence of any individual complication was not determined [147].

Because of its consistent morbidity, after successful conservative management, the routine indication to interval appendectomy is justified only in case of persistent or recurrent symptoms, and should be avoided in asymptomatic patients [148]. Some authors recommend routine interval appendectomy, not to avoid the risk of recurrence, but to rule out possible appendicular neoplasia. In the retrospective study by Carpenter et al., including 315 patients with AA, 18 out of 24 patients with complicated appendicitis (7.6 % of the total series) that were treated conservatively, underwent interval appendectomy. The incidence of neoplasms was significantly higher in the patients underwent interval appendectomy than in the immediate appendectomy group (five patients, 28 % vs. three patients, 1 % *P* < 0.0001). Appendicular or colonic neoplasms should be investigated after nonoperative management of AA, especially in patients older than 40 years [149].

**Statement 7.1: Percutaneous drainage of a periappendicular abscess, if accessible, is an appropriate treatment in addition to antibiotics for complicated appendicitis. (EL 2, GOR B)**

**Statement 7.2: Non-operative management is a reasonable first line treatment for appendicitis with phlegmon or abscess. (EL 1, GOR A)**

**Statement 7.3: Operative management of acute appendicitis with phlegmon or abscess is a safe alternative to non-operative management in experienced hands. (EL 2, LOR B)**

**Statement 7.4: Interval appendectomy is not routinely recommended both in adults and children. (EL 1, LOR A)**

**Statement 7.5: Interval appendectomy is recommended for those patients with recurrent symptoms. (EL 2, LOR B)**

**Statement 7.6: Colonic screening should be performed in those patients with appendicitis treated non-operatively if >40y/o. (EL 3, LoR C)**

### Preoperative and postoperative antibiotics

*Should Preoperative antibiotics prophylaxis be given? What antibiotics? When should postoperative antibiotics be given? What antibiotics? Duration? (Speaker in Jerusalem CC Dr. M. Sartelli)*

In the last years use of antibiotics in patients undergoing appendectomy has been debated [150, 151].

In 2005 a Cochrane meta-analysis supported that broad-spectrum antibiotics given preoperatively are effective in decreasing wound infection and abscesses. Randomised Controlled Trials (RCTs) and Controlled Clinical Trials (CCTs) in which any antibiotic regime were compared to placebo in patients suspected of having appendicitis, and undergoing appendectomy were analysed. Forty-five studies including 9576 patients were included in this review. Antibiotics were superior to placebo for preventing wound infection and intra-abdominal abscess, with no apparent difference in the nature of the removed appendix [152].

In 2005 a randomized controlled trial on 269 patients, aged 15–70 years, with non-perforated appendicitis undergoing open appendectomy was published. 92 patients received single dose preoperative (group A), 94 received three-dose (group B) and 83 received 5-day perioperative (group C) regimens of cefuroxime and metronidazole. The rate of postoperative infective complication was not significantly different among the groups (6.5 % group A, 6.4 % group B, 3.6 % group C). The duration of antibiotic therapy had no significant effect on the length of hospital stay. Complications related to antibiotic treatment were significantly more common for 5-day perioperative antibiotic group (C) compared with single dose preoperative antibiotic group (A) (*P* = 0.048) [153].

Some prospective trials demonstrated that patients with perforated appendicitis should have postoperative antibiotic treatment [154, 155]. The major pathogens involved in community-acquired appendicitis are Enterobacteriaceae, Streptococcus species, and anaerobes (especially *B. fragilis*) [156].

In 2013 the World Society of Emergency Surgery published their guidelines for management of intra-abdominal infections (IAIs) stratifying the antimicrobial regimen according to patient’s condition (Sepsis Vs. severe sepsis and septic shock), the pathogens presumed to be involved, and the risk factors indicative of major resistance patterns [157].

Many studies compared duration of antibiotic regimens for perforated appendicitis and they showed a variation in the duration of treatment [154, 155, 158].

In 2000 Taylor et al. published a prospective trial comparing a minimum IV 5-days antibiotic regimen versus no minimum IV regimen. Infectious complications were not statistically different between the two groups. Average hospital stay was also not statistically different between the two groups. The study demonstrated that an antimicrobial regimen with no minimum IV antibiotic requirement in patients with complicated appendicitis did not increase morbidity. Furthermore, the protocol arm with no minimum IV antibiotic requirement led to less IV antibiotic use but did not significantly decrease hospital stay [159].

Recently, a prospective randomized trial on 518 patients with complicated intra-abdominal infection, including also complicated appendicitis, undergoing adequate source control demonstrated the outcomes after fixed-duration antibiotic therapy (approximately 4 days) were similar to those after a longer course of antibiotics (approximately 8 days) that extended until after the resolution of physiological abnormalities [160].

Although discontinuation of antimicrobial treatment should be based on clinical and laboratory criteria, a period of 3–5 days for adult patients is generally sufficient to treat complicated acute appendicitis.

**Statement 8.1: In patients with acute appendicitis preoperative broad-spectrum antibiotics are always recommended. (EL 1, GoR A)**

**Statement 8.2: For patients with uncomplicated appendicitis, post-operative antibiotics are not recommended .(EL 2, GoR B)**

**Statement 8.3: In patients with complicated acute appendicitis, postoperative, broad-spectrum antibiotics are always recommended. (EL 2, GoR B)**

**Statement 8.4: Although discontinuation of antimicrobial treatment should be based on clinical and laboratory criteria such as fever and leucocytosis, a period of 3–5 days for adult patients is generally recommended. (EL 2, GoR B)**

## Conclusions

The current evidence-based Guidelines represent to the best of our knowledge, the first international Comprehensive Clinical Guidelines for diagnosis and management of Acute Appendicitis. During the 3rd World Congress of the WSES, held in Jerusalem (Israel) in July 2015, a panel of experts including an Organizational Committee and Scientific Committee and Scientific Secretariat, participated to a Consensus Conference where eight panelists (SDS, MDK, FC, DW, MiSu, MaSa, MDM, CAG) presented a number of statements, which were developed for each of the eight main questions about diagnosis and management of AA (Appendix). The statements were then voted, eventually modified and finally approved by the participants to The Consensus Conference and subsequently by the board of co-authors. The current paper is reporting the definitive Guidelines Statements and Clinical Recommendations on each of the following topics: 1) Diagnostic efficiency of clinical scoring systems, 2) Role of Imaging, 3) Non-operative treatment for uncomplicated appendicitis, 4) Timing of appendectomy and in-hospital delay, 5) Surgical treatment 6) Scoring systems for intra-operative grading of appendicitis and their clinical usefulness 7) Non-surgical treatment for complicated appendicitis: abscess or phlegmon 8) Pre-operative and post-operative antibiotics. In summary, The Alvarado score (with cut-off score < 5) is sufficiently sensitive to exclude acute appendicitis, nonetheless the ideal (highly sensitive and specific), clinically applicable, diagnostic scoring system/clinical rule remains currently out of reach. Imaging should be linked to Risk Stratification such as AIR or Alvarado score, low-risk patients being admitted to hospital and not clinically improving or reassessed score could have appendicitis ruled in or out by abdominal CT, in high-risk and young preoperative imaging may be avoided, MRI is recommended in pregnant patients with suspected appendicitis. Regarding non-operative treatment of AA, antibiotic therapy can be successful in selected patients with uncomplicated appendicitis who wish to avoid surgery and accept the risk up to 38 % recurrence. The timing of performing an appendectomy is a great matter of debate and our recommendations are that a short, in-hospital surgical delay up to 12/24 h is safe in uncomplicated acute appendicitis and does not increase complications and/or perforation rate, however surgery for uncomplicated appendicitis should be planned for next available list minimizing delay wherever possible. When analysing the surgical treatment, laparoscopic appendectomy should represent the first choice where laparoscopic equipment and skills are available, since it offers clear advantages in terms of less pain, lower incidence of SSI, decreased LOS, earlier return to work and overall costs. In particular, laparoscopy offers clear advantages and should be preferred in obese patients, older patients and patients with comorbidities. In experienced hands, laparoscopy is more beneficial and cost-effective than open surgery for complicated appendicitis. Laparoscopy should not be considered as a first choice over open appendectomy in pregnant patients. No major benefits have also been observed in laparoscopic appendectomy in children, but it reduces hospital stay and overall morbidity. Analysing the technical issues in performing an appendectomy, peritoneal irrigation does not have any advantages over suction alone in complicated appendicitis; there are no clinical differences in outcomes, LOS and complications rates between the different techniques described for mesentery dissection (monopolar electrocoagulation, bipolar energy, metal clips, endoloops, Ligasure, Harmonic Scalpel etc.). There are no clinical advantages in the use of endostapler over endoloops for stump closure for both adults and children, but Endoloops might be preferred for lowering the costs when appropriate skills/learning curve are available. Finally, drains are not recommended in complicated appendicitis in paediatric patients, in adult patients, drain after appendectomy for perforated appendicitis and abscess/peritonitis should be used with judicious caution, given the absence of good evidence from the literature. Drains did not prove any efficacy in preventing intra-abdominal abscesses and seem to be associated with delayed hospital discharge.

Delayed primary skin closure does not seem beneficial for reducing the risk of SSI and increase LOS in open appendectomies with contaminated/dirty wounds. When a “normal” looking appendix is found at surgery and no other disease is found in a symptomatic patient, we recommend its removal. Percutaneous drainage of a periappendiceal abscess, if accessible, is an appropriate treatment in addition to antibiotics for complicated appendicitis. Non-operative management is a reasonable first line treatment for appendicitis with phlegmon or abscess. Operative management of acute appendicitis with phlegmon or abscess can be a safe alternative to non-operative management but only in experienced hands. Interval appendectomy is not routinely recommended both in adults and children, but it can be recommended for those patients with recurrent symptoms. Important is to recommend colonic screening in patients >40 y/o with appendicitis treated non-operatively. Finally, in patients with acute appendicitis preoperative broad spectrum antibiotics are recommended, for patients with uncomplicated appendicitis postoperative antibiotics are not recommended, whereas in those with complicated acute appendicitis postoperative, broad spectrum antibiotics are always recommended, usually for a period of 3–5 days.

After reaching consensus on each of the above mentioned statements proposed by every one of the Speakers of the Panel (see Appendix), the participants to the Consensus Conference in Jerusalem and the Scientific Committee members, developed and shared the WSES algorithm for diagnosis and management of Acute Appendicits, reported in Fig. 1.

## Abbreviations

AA, acute appendicitis; AAS score, Adult Appendicitis Score; AIR, Appendicitis Inflammatory Response Score; AS, Alvarado Score; ASA, American Society of Anaesthesiology; CC, Consensus Conference; CCT, Controlled Clinical Trials; CT, computed tomography; GoR, grade of recommendation; IAA, Intra-abdominal abscess; LA, Laparoscopic Appendectomy; LoE or EL, level of evidence; LOS, length of stay; MRI, magnetic resonance imaging; OA, open appendectomy; OC, Organization Committee; OR, odds ratio; POR, pooled odds ratio; RCT, randomised controlled trials; RIPASA score, Raja Isteri Pengiran Anak Saleha Appendicitis; SC, Scientific Committee; SD, standard deviation; SS, Scientific Secretariat; SSI, surgical site infection; US, ultrasound; WSES, World Society of Emergency Surgery; RIF, right iliac fossa

## Appendix

**Table 3:** Guidelines Statements

	LE	GoR	Statement
1) Diagnostic efficiency of clinical scoring systems			
1.1	1	A	The Alvarado score (with cutoff score < 5) is sufficiently sensitive to exclude acute appendicitis.
1.2	1	A	The Alvarado score is not sufficiently specific in diagnosing acute appendicitis.
1.3	1	B	An ideal (high sensitivity and specificity), clinically applicable, diagnostic scoring system/clinical rule remains outstanding. This remains an area for future research
2) Role of imaging			
2.1	2	B	In patients with suspected appendicitis a tailored individualised approach is recommended, depending on disease probability, sex and age of the patient
2.2	2	B	Imaging should be linked to Risk Stratification such as AIR or Alvarado score
2.3	2	B	Low risk patients being admitted to hospital and not clinically improving or reassessed score could have appendicitis ruled-in or out by abdominal CT
2.4	2	B	Intermediaterisk classification identifies patients likely to benefit from observation and systematic diagnostic imaging.
2.5	2	B	Highrisk patients (younger than 60 yearsold) may not require preoperative imaging.
2.6	3	B	US Standard reporting templates for ultrasound and US three step sequential positioning may enhance over accuracy .
2.7	2	B	MRI is recommended in pregnant patients with suspected appendicitis, if this resource is available
3) Nonoperative treatment for uncomplicated appendicitis			
3.1	1	A	Antibiotic therapy can be successful in selected patients with uncomplicated appendicitis who wish to avoid surgery and accept the risk up to 38 % recurrence.
3.2	2	B	Current evidence supports initial intravenous antibiotics with subsequent conversion to oral antibiotics.
3.3	2	B	In patients with normal investigations and symptoms unlikely to be appendicitis but which do not settle: • Cross-sectional imaging is recommended before surgery • Laparoscopy is the surgical approach of choice • There is inadequate evidence to recommend a routine approach at present
4) Timing of appendectomy and in-hospital delay			
4.1	2	B	Short, in-hospital surgical delay up to 12/24 h is safe in uncomplicated acute appendicitis and does not increase complications and/or perforation rate.
4.2	2	B	Surgery for uncomplicated appendicitis can be planned for next available list minimizing delay wherever possible (patient comfort etc.).
5) Surgical treatment			
5.1.1	1	A	Laparoscopic appendectomy should represent the first choice where laparoscopic equipment and skills are available, since it offers clear advantages in terms of less pain, lower incidence of SSI, decreased LOS, earlier return to work and overall costs.
5.1.2	2	B	Laparoscopy offers clear advantages and should be preferred in obese patients, older patients and patients with comorbidities
5.1.3	2	B	Laparoscopy is feasible and safe in young male patients although no clear advantages can be demonstrated in such patients.
5.1.4	1	B	Laparoscopy should not be considered as a first choice over open appendectomy in pregnant patients
5.1.5	1	A	No major benefits have also been observed in laparoscopic appendectomy in children, but it reduces hospital stay and overall morbidity
5.1.6	3	B	In experienced hands, laparoscopy is more beneficial and cost-effective than open surgery for complicated appendicitis
5.2	2	B	Peritoneal irrigation does not have any advantages over suction alone in complicated appendicitis
5.3.1	3	B	There are no clinical differences in outcomes, LOS and complications rates between the different techniques described for mesentery dissection (monopolar electrocoagulation, bipolar energy, metal clips, endoloops, Ligasure, Harmonic Scalpel etc.).
5.3.2	3	B	Monopolar electrocoagulation and bipolar energy are the most cost-effective techniques, even if more experience and technical skills is required to avoid potential complications (e.g. bleeding) and thermal injuries.
5.4.1	1	A	There are no clinical advantages in the use of endostapler over endoloops for stump closure for both adults and children
5.4.2	3	B	Endoloops might be preferred for lowering the costs when appropriate skills/learning curve are available
5.4.3	2	B	There are no advantages of stump inversion over simple ligation, either in open or laparoscopic surgery
5.5.1	3	B	Drains are not recommended in complicated appendicitis in paediatric patients
5.5.2	1	A	In adult patients, drain after appendectomy for perforated appendicitis and abscess/peritonitis should be used with judicious caution, given the absence of good evidence from the literature. Drains did not prove any efficacy in preventing intraabdominal abscess and seem to be associated with delayed hospital discharge.
5.6	1	A	Delayed primary skin closure does not seem beneficial for reducing the risk of SSI and increase LOS in open appendectomies with contaminated/dirty wounds
6) Scoring systems for intraoperative grading of appendicitis and their clinical usefulness			
6.1	2	B	The incidence of unexpected findings in appendectomy specimens is low but the intraoperative diagnosis alone is insufficient for identifying unexpected disease. From the current available evidence, routine histopathology is necessary
6.2	4	C	There is a lack of validated system for histological classification of acute appendicitis and controversies exist on this topic.
6.3	2	B	Surgeon’s macroscopic judgement of early grades of acute appendicitis is inaccurate
6.4	4	C	If the appendix looks “normal” during surgery and no other disease is found in symptomatic patient, we recommend removal in any case.
6.5	2	B	We recommend adoption of a grading system for acute appendicitis based on clinical, imaging and operative findings, which can allow identification of homogeneous groups of patients, determining optimal grade disease management and comparing therapeutic modalities
7) Nonsurgical treatment for complicated appendicitis :abscess or phlegmone			
7.1	2	B	Percutaneous drainage of a periappendiceal abscess, if accessible, is an appropriate treatment in addition to antibiotics for complicated appendicitis.
7.2	1	A	Nonoperative management is a reasonable first line treatment for appendicitis with phlegmon or abscess
7.3	2	B	Operative management of acute appendicitis with phlegmon or abscess is a safe alternative to nonoperative management in experienced hands
7.4	1	A	Interval appendectomy is not routinely recommended both in adults and children.
7.5	2	B	Interval appendectomy is recommended for those patients with recurrent symptoms.
7.6	3	C	Colonic screening should be performed in those patients with appendicitis treated non-operatively if >40y/o
8) Preoperative and Postoperative Antibiotics			
8.1	1	A	In patients with acute appendicitis preoperative broad-spectrum antibiotics are always recommended
8.2	2	B	For patients with uncomplicated appendicitis, postoperative antibiotics are not recommended
8.3	2	B	In patients with complicated acute appendicitis, postoperative, broad-spectrum antibiotics are always recommended
8.4	2	B	Although discontinuation of antimicrobial treatment should be based on clinical and laboratory criteria such as fever and leucocytosis, a period of 3–5 days for adult patients is generally recommended
